# The Long-Term Effects of a Low–Fermentable Oligosaccharides, Disaccharides, Monosaccharides, and Polyols Diet for Irritable Bowel Syndrome Management

**DOI:** 10.1016/j.cdnut.2023.101997

**Published:** 2023-09-06

**Authors:** Julie A. Bardacke, Linda Yarrow, Sara K. Rosenkranz

**Affiliations:** 1 Department of Food, Nutrition, Dietetics and Health, College of Health and Human Sciences, Kansas State University, Manhattan, KS, United States; 2 Department of Kinesiology and Nutrition Sciences, College of Integrated Health Sciences, University of Nevada Las Vegas, Las Vegas, NV, United States

**Keywords:** low-FODMAP diet (LFD), irritable bowel syndrome (IBS), IBS management, IBS treatment, digestive disorders, gut-brain disorders, diet therapy

## Abstract

Short-term studies indicate that low–fermentable oligosaccharides, disaccharides, monosaccharides, and polyols diets (LFDs) can improve symptoms for patients with irritable bowel syndrome (IBS). However, long-term (≥6 mo) effectiveness, safety, and sustainability of an LFD are not well understood and remain controversial. The primary purpose of the current review was to consider the published research on the effectiveness, safety, and sustainability of an LFD for patients with IBS. The secondary aim was to develop an infographic for dissemination to outpatient registered dietitian nutritionists and other healthcare professionals who work with patients with IBS. Three electronic databases (PubMed, Scopus, and Web of Science) were searched through December 2022, using the terms irritable bowel syndrome, FODMAP, and long-term. Following article selection, a total of 14 studies were included. Nine of 9 studies reported significant improvements in symptoms, 7 of 7 studies showed significant improvements in bowel habits, 1 of 1 study showed significantly improved disease course, and 6 of 6 studies showed significantly improved quality of life, compared to baseline. One study showed that improvement in gastrointestinal symptoms was significantly correlated with improvements in quality of life. Two of 3 studies and body composition measures indicated that nutritional adequacy was not compromised. Two of 2 studies showed that gut microbiota did not change, but 1 study showed decreased short-chain fatty acids. Adherence rates ranged from 50% to 82%, and 1 study showed that greater adherence was significantly correlated with improved IBS symptoms. Three of 3 studies showed that better adherence to an LFD was associated with improved symptom relief, and 70%–89% of participants reported satisfaction with the LFD for IBS management. The main difficulties reported were the higher expense and adhering to the diet when eating at restaurants, with family and friends, or while traveling. Overall, a long-term LFD for IBS management can be effective, safe, and sustainable.

## Introduction

Irritable bowel syndrome (IBS) is a prevalent gastrointestinal (GI) disorder affecting 1 in 7 adults and is characterized by chronic, relapsing symptoms without overt abnormal pathology [1]. Common IBS symptoms include lower abdominal pain and discomfort, bloating, flatulence, distension, diarrhea and/or constipation, among others [1]. Having IBS is a lifelong chronic condition with fluctuating symptom patterns, symptom severity, and disease course. The pathophysiology of IBS is still not completely understood but might be influenced by dietary intolerances, genetics, chronic low-grade inflammation, anxiety and depression, and poor lifestyle habits [2]. Having IBS can greatly impact patient quality of life (QOL), work productivity, and healthcare utilization. Diagnosis of IBS currently uses the Rome IV criteria, which was recently updated from the Rome III criteria, and now has criteria with more severe symptoms than the Rome III criteria [3]. IBS is categorized into 4 main subgroups: diarrhea-predominant, constipation-predominant, mixed or alternating diarrhea and constipation, or unclassified IBS, which has its own diagnostic criteria based upon the Bristol stool chart (BSC) [4].

There are many different types of treatments for IBS, such as medications like chloride channel activators and guanylate cyclase activators to treat global IBS with constipation symptoms, therapy such as gut-directed psychotherapy, and diet and lifestyle changes [5]. Treatment should be individualized to the patient and according to the IBS subclassification. The benefits from prolonged treatments must be compared with the potential negative effects and the risks of continued treatment. An estimated 60%–80% of patients with IBS report that their symptoms are food-related, and over half of the gastroenterologists in the United States recommend diet therapy to more than 75% of their patients with IBS [2,6]. Recommended diets include general IBS dietary advice, low–fermentable oligosaccharides, disaccharides, monosaccharides, and polyols (LFD), gluten-free, and lactose-free diets. While there is limited evidence to support LFD, particularly over the long term, the general IBS dietary advice is used as a first-line dietary therapy, after which an LFD is recommended if IBS symptoms persist [7]. General IBS dietary and lifestyle advice includes following regular eating patterns, reducing insoluble fiber, alcohol, caffeine, spicy foods, and fats, as well as increasing exercise and hydration [7]. A growing body of evidence shows that an LFD provides symptomatic benefits in about 50%–80% of patients with IBS and is recommended as a first-line dietary therapy for IBS in many countries [8].

An LFD involves a reduction in total fermentable oligosaccharides, disaccharides, monosaccharides, and polyols (FODMAP) intake per serving [1,9]. FODMAPs are short-chained carbohydrate compounds that are not well-absorbed by the small intestine and are found in a wide variety of foods. When these foods move into the colon, they osmotically pull fluid into the intestinal lumen, while the colonic bacteria ferment the unabsorbed FODMAPs [10]. Both mechanisms can trigger IBS symptoms such as bloating, cramping, flatulence, and diarrhea. In addition, some research suggests that FODMAPs might increase endothelial barrier permeability, influence colonic microbiota, cause immune activation and low-grade inflammation, all of which may influence IBS pathology [9].

Administering an LFD involves 3 phases: a strict LFD phase, a reintroduction phase, and a personalization phase [1]. The strict LFD phase typically lasts 2–6 wk to induce symptom control. The reintroduction phase lasts 8–12 wk and involves food challenges of varying durations to identify individual FODMAP sensitivities. The personalization phase has no time limit and is used to establish a minimally restrictive but effective long-term diet [1]. Despite the research showing short-term benefits, there are concerns and questions surrounding the difficulty and complexity of teaching, learning, and following this diet, as well as its long-term nutritional adequacy and any potential effects on the microbiome. Registered dietitian nutritionists (RDNs) are recognized as the main educators of the LFD diet [11], and share concerns regarding a long-term LFD, in part because of the increasing nonrecommended use of this type of diet for weight loss.

The primary aim of the current study was to review the available published research on the effectiveness, safety, and sustainability of a long-term LFD for patients with IBS. The secondary aim was to synthesize these review results to develop an infographic for dissemination to outpatient RDNs and other healthcare professionals who work with patients with IBS. By providing guidance that can be quickly incorporated into professional practice, this information may lead to improved IBS management.

## Methods

A search for relevant studies was conducted through December 2022 in 3 online electronic databases: PubMed, Scopus, and Web of Science. The searches included the following keywords: “irritable bowel syndrome,” “FODMAP,” and “long term.” Searches were limited to articles available in English and on adult humans. Studies where interventions were <6 mo in duration were excluded. In total, searches yielded 205 records. Of the 205 records, 110 were duplicates. Of the remaining 95 articles, 85 were excluded based on title, abstract relevance, type of study, or study duration. Four additional studies were identified through hand-searching reference lists of the included articles. A total of 14 primary research studies met all selection criteria and were reviewed to identify the long-term effects of an LFD in patients with IBS. Study population characteristics and methodology data were extracted and summarized by study design and duration, percentage of participants who completed the study compared with those initially enrolled, participant characteristics, the number of times and when LFD evaluations with questionnaires were given to participants, the type of dietary educator, and the number of dietary consultations and when they were conducted during the study. Results were categorized and synthesized into 3 broad areas: *1*) effectiveness (symptom relief, QOL); *2*) safety (dietary intake, weight and body composition, microbiota); *3*) and sustainability (adherence, acceptance). QOL includes data about possible changes in healthcare usage (i.e., number of doctor visits pertaining to IBS), medication usage for IBS, and absence from work due to IBS symptoms. The criteria for data in the “safety” category would be any data that show how the diet could affect the physical health of the participants. Based on what types of data were gathered in the included studies, 3 general subcategories of dietary intake, weight and body composition, and microbiota were identified. Dietary intake measured the nutritional adequacy of diet at long-term follow-up by comparing the energy, macronutrient (including fiber), and micronutrient intake to nationally recommended values (i.e., Dietary Reference Values in the UK or the Estimated Average Requirement in New Zealand). Weight and body composition data included any significant physiological changes, excluding the microbiota, between baseline and long-term follow-up. Microbiota data included any significant changes in microbiota or metabolic products resulting from the diet, between baseline and long-term follow-up. This critical review culminated in a product (infographic) arising from the synthesis of the broad range of data categories included in the studies encompassed in the review.

## Results

### Study population characteristics and methodologies

As shown in Table 1, 7 of the 14 studies were prospective cohort studies, 4 were retrospective cross-sectional studies, 1 was a randomized clinical trial without a control, and 2 were randomized controlled trials (RCTs). Results did not display patterns based on study design such as RCT compared with retrospective studies. All studies included patients who had been diagnosed with IBS. Common exclusion criteria included comorbidity with other major diseases or illnesses, major abdominal surgery, taking other medications that affected abdominal pain, pregnancy, or lactation, previously on LFD, drug/alcohol abuse, psychiatric disease, language barriers, unable to give written informed consent, failing to attend treatment, and nonresponse. Eight studies included patients with all IBS subtypes, whereas the remaining 6 excluded some subtypes or did not list subtype information. The study durations ranged from 6 mo to 4 y. The percentage of participants with IBS who completed the long-term evaluations, out of those initially enrolled, ranged from 19% to 83%, and the completion rate was not associated with any type of study or IBS subtype. Participants dropped out during studies due to difficulty adhering to the diet, unsatisfactory symptom response to the LFD, onset of intercurrent disease or need for surgery, change of job/address, return to habitual diet (HD) due to well-being, or withdrawal due to lack of time or a personal reason. Participants were excluded from analyses due to incorrectly or incompletely filled-out questionnaires.

**TABLE 1:** Study demographics and methodology

Study	Study design	Study duration	Diagnostic criteria	No. of participants initially enrolled to receive LFD treatment	No. of participants evaluated at long-term follow-up (%compared to initially enrolled)	Participant age and sex	Participant IBS subtype	LFD questionnaire evaluations (no. and time points)	Dietary educator type	No. of dietary consultations and time points
Ankersen et al. [30], 2021	Open-label randomized double-crossover trial (RCT)	12 mo	Rome III	21	Specific number of participants in LFD group analyzed at long-term follow-up not provided	Baseline data for both LFD and probiotic treatment groups (*n* = 34): F 23 (68%), M 11 (32%), median age 44.5 y. (range: 27.5–51.5)	Baseline data for both LFD and probiotic treatment groups (*n* = 34): IBS-M 15 (44%), IBS-D 19 (56%)	3 evaluations using information from IBS CC web application: baseline, reintroduction phase at 4 wk, and at 12 mo	Nutritionist	2 consults: initial and 4 wk. For the reintroduction phase
Bellini et al. [21], 2020	Prospective cohort	6–24 mo	Rome IV	73	41 (56%)	Baseline (*n* = 73): F 64 (87%), M 9 (12%), mean age 45.27 y Long-term follow-up (*n* = 41): F 37(90%), M 4 (10%), mean age 45.63 y	Subtype data not provided	4 evaluations: baseline, reintroduction phase at 8wk, before AdLFD phase, 6–24 mo	Nutritionist	3+ consults: initial, 8 wk, starting AdLFD phase, on demand
De Roest et al. [15], 2013	Prospective cohort	∼15.7 mo (±9.0 mo)	Diagnostic criteria not provided	Not applicable	90	Eligible participants that replied (*n* = 90): F 76 (84%), M 14 (16%) mean age 47.0 y (SD 15.3)	Subtype data not provided	3 evaluations: baseline, 6 wk., and at follow-up (∼15.7 mo)	Dietitian	2 consults: initial and 6 wk. For the reintroduction phase
Gravina et al. [19], 2020	Prospective cohort	∼10.5 mo	Rome IV	120	100 (83%)	Enrolled participants (*n* = 120): F 72 (60%), M 48 (40%) Long-term follow-up (*n* = 100): F 60 (60%), M 40 (40%)	Enrolled participants (*n* = 120): IBS-C 24 (20%), IBS-D 28 (23%), IBS-M 68 (57%) Long-term follow-up (*n* = 100): IBS-C 20 (20%), IBS-D 24 (24%), IBS-M 56 (56%)	4 evaluations: baseline, 6 wk, 4.5 mo, 10.5 mo	GI doctor	Unknown number of consults
Harvie et al. [16], 2017	Nonblinded RCT	6 mo	Rome III	50 (divided into 2 parallel groups)	34 (68%)	Group 1 (*n* = 23): F 17 (73%), M 6 (26%), mean age 43.3 y Group 2 (*n* = 27): F 26 (96%), M 1 (4%), mean age 40.6 Participants at final evaluation (*n* = 34): demographic data not provided	Group 1 (*n* = 23): IBS-D 16 (69%), IBS-C 3 (13%), IBS-M 5 (22%) Group 2 (*n* = 27): IBS-D 16 (59%), IBS-C 2 (9%), IBS-M 9 (33%) Participants at final evaluation (*n* = 34): subgroup data not provided	3 evaluations for both groups: baseline, reintroduction phase at 3 mo, and 6 mo	Dietitian	2+ consults: initial, 3 mo. For the reintroduction phase, and on demand
Kortlever et al. [17], 2019	Prospective cohort	6 mo	Rome III	101	56 (55%)	Baseline (*n* = 101 enrolled): 84 (83%) F, mean age 41.9 y (SD 16.0) At 6 mo (*n* = 56): F 51 (91%), M 5 (9%), mean age 45.6 y (SD 16.0)	Baseline (*n* = 101): IBS-C 12 (12%), IBS-D 42 (41%), IBS-U 4 (4%), IBS-M 44 (43%) At 6 mo (*n* = 56): IBS-C 4 (7%), IBS-D 25 (45%), IBS-U 1 (2%), IBS-M 26 (46%)	3 evaluations: baseline, reintroduction phase at 6 wk, and at 6 mo	Dietitian	2 consults: initial and 6 wk. For the reintroduction phase
Maagaard et al. [10], 2016	Retrospective cross-sectional	∼16 mo	Rome III	Not applicable	131	Participants that responded long-term (*n* = 131): 107 (82%) F, 24 (18%) M, median age 43 (range 18–85)	Participants that responded long-term (*n* = 131): IBS-D 47 (40%), IBS-C 44 (37%), IBS-M 17 (14%), IBS-U 10 (9%)	1 evaluation: ∼16 mo. After initial consult, no baseline data	Dietitian	2 consults: initial and 6–8 wk For the reintroduction phase
Nawawi et al. [20], 2020	Retrospective cross-sectional	12 mo	Rome IV	Not applicable	41	Baseline study cohort not enrolled (*n* = 127): F 108 (85%), M 19 (15%), median age 45 y (range 16–80)	Baseline study cohort not enrolled (*n* = 127): IBS-D 58, IBS-C 41	1 evaluation: 12 mo. After the initial consult, compared with existing baseline data	Dietitian	3+ consults: initial, at 2–3 mo. For the reintroduction phase, every 3 mo
O’Keeffe et al. [12], 2018	Prospective cohort	6–18 mo	NICE	143	103 (72%)	Long-term follow-up (*n* = 103): F 76 (74%), M 27 (26%), mean age 49 (SD 15)	Long-term follow-up (*n* = 103): IBS-C 20 (19%), IBS-D 39 (38%), IBS-M 21 (20%), IBS-U 23 (22%)	3 evaluations: baseline reintroduction phase at 6 wk, and 12–24 wk	Dietitian	2 consults: initial and at 6 wk. For the reintroduction phase
Peters et al. [13], 2016	Randomized clinical trial without a control	6 mo	Rome III	24	Unknown number of participants in LFD treatment group that completed long-term follow-up	Participants enrolled and randomly assigned into LFD treatment group (*n* = 24): F 19 (79%), M 5 (36%), median age 34 (range 23–66)	Participants enrolled and randomly assigned into LFD treatment group (*n* = 24): IBS-D 10 (42%), IBS-C 5 (21%), IBS-M 9 (37%)	3 evaluations: baseline, 6 wk, and at 6 mo	Dietitian	2 consults: initial and 6 wk. For the reintroduction phase
Rej et al. [2], 2021	Retrospective cross-sectional	∼3.7 y	Rome III	Not applicable	205	Participants completed long-term study (*n* = 205): F 153 (75%), M 52 (25%), mean age 50 ± 16 y	Participants completed long-term study (*n* = 205): IBS-D 101 (49%), IBS-M 24 (12%), IBS-C 31 (15%), IBS-U 46 (22%)	1 evaluation: >6 mo. After the initial consult, only 36% of participants had baseline data to compare	Dietitian	2 consults: initial and at the start of the reintroduction phase
Seamark et al. [18], 2021	Prospective cohort	12–16 mo	Diagnosed by GP, per the local diagnosis pathway	Not applicable	211	Long-term follow-up (*n* = 211): F 182 (86%), mean age 53.6 y (SD 15)	Subtypes data not provided	3 evaluations: baseline, reintroduction phase at 4–8 wk, and ∼12 mo	Dietitian	2 consults: initial and 4–8 wk. For the reintroduction phase
Staudacher et al. [14], 2022	Prospective cohort	12 mo	Rome III	95	18 (19%)	Long-term follow-up (*n* = 18): F 11 (61%), M 7 (39%), median age 33 y (SD 20)	Long-term follow-up (*n* = 18): 14 (78%) IBS-D, 2 (11%) IBS-M, 2 (11%) IBS-U	1 evaluation: 12 mo, compared with baseline data	Dietitian	2 consults: initial and at 4–8 wk. For the reintroduction phase
Weynants et al. [22], 2020	Retrospective cross-sectional	1–3 y	Rome III	Not applicable	90	Participants analyzed (*n* = 90): F 65 (79%), M 17 (19%), 8 missing, Median age 41.5 y (range 17–69)	Participants analyzed (*n* = 90): IBS-U 4 (5%), IBS-D 32 (39%), IBS-C 8 (10%), 38 (46%), missing 8	1 evaluation: 1–3 y after the initial consult, no baseline data	Dietitian	3+ consults: initial, 6–8 wk. For the reintroduction phase, at 18–20 wk. To start the AdLFD phase, on demand

Abbreviations: AdLFD, adjusted low–fermentable oligosaccharides, disaccharides, monosaccharides, and polyols diet phase; F, female; GI, gastrointestinal; GP, General Practitioner; IBS-C, irritable bowel syndrome with constipation; IBS-D, irritable bowel syndrome with diarrhea; IBS-M, irritable bowel syndrome mixed; IBS-U, irritable bowel syndrome undefined; LFD, low–fermentable oligosaccharides, disaccharides, monosaccharides, and polyols diet; M, male; NICE, National Institute of Clinical Excellence; RCT, randomized controlled trial; Rome III, diagnostic criteria provides criteria for diagnosis of irritable bowel syndrome updated from the Rome II criteria; Rome IV, diagnostic criteria provides criteria for diagnosis of irritable bowel syndrome updated from the Rome III criteria.

LFD questionnaire evaluations were typically completed at baseline and at the beginning of phase changes. Nine studies had 3 or 4 evaluations, whereas 5 studies, which were mostly retrospective studies, had 1 evaluation. Data collected were either compared with baseline and/or to other patients who had returned to their HD. Two retrospective studies did not include baseline data in the results. Nine studies included 3 phases of the LFD or were follow-ups to studies that included all 3 phases, whereas 5 studies only included the first 2 phases. The duration of the first phase in these studies ranged from 4 to 12 wk, with details about the LFD advice often provided. Although the reintroduction phase duration and details were provided in a few studies, the personalization phase was not detailed in any of the studies.

Eleven of 14 studies included RDNs as the dietary educator, 2 studies included nutritionists, and 1 study included a GI doctor. Eleven of 14 studies provided 2 dietary consultations, 1 at the start of the strict phase and one at the start of the reintroduction phase. Two of these 11 offered additional support outside of consultations [2,10,[12], [13], [14], [15], [16], [17], [18], [19]]. Many of these studies did not distinctly address the personalization phase, instead educating on the latter 2 phases in 1 consultation. Three studies provided 3 consultations, 1 at the beginning of each phase, with additional on-demand support [[20], [21], [22]].

### Effectiveness

Effectiveness was measured by assessing symptom relief and QOL.

#### Symptom relief

Symptom relief was measured both by changes in GI symptoms and disease course. The different tests used to assess these outcomes are described below and in Table 2.

**TABLE 2:** Effectiveness

Study	Symptom relief	QOL
Ankersen et al. [30], 2021	IBS-SSS: significant decreases for LFD responders relative to nonresponders at long-term follow-up (*P* < 0.05), and post hoc analysis suggests that sustained long-term symptom control could be obtained relative to patients’ baseline BSC and bowel movement frequency: no significant changes in bowel movements per day based on the responder type were observed. Unclear if long term compared to baseline Copenhagen disease course: at baseline, none of the patients with IBS reported an indolent disease course type (i.e., type A), but at 1-y follow-up, 42% (5/12) of the LFD responder reported a type A. However, study does not indicate if this is significant improvement. None of LFD nonresponders reported type A at 1-y follow-up	IBS-QOL: the corresponding changes in QOL among LFD responders was not significant compared to nonresponders, but post hoc models of QOL showed that LFD responders had significant increases in QOL while on the LFD, and even more during reintroduction relative to their baseline QOL (no *P* value provided)
Bellini et al. [21], 2020	IBS-SSS: significantly improved for 34/41 patients from baseline to follow-up (*P* < 0.001) Bowel habits questionnaire: incomplete evacuation, soft stools, defecatory urgency, abdominal pain, and bloating significantly improved from baseline to long-term follow-up (all *P* < 0.05). Straining at defecation, painful defecation, hard stools, fragmented defecation, and gas/feces incontinence did not significantly improve from baseline to long-term follow-up (ns)	SF-36: pain, vitality, social activity, physical and mental health indexes significantly improved from baseline (all *P*s < 0.05). Physical activity, physical and emotional role limitations, mental health, and general health did not significantly change (ns) Hospital Anxiety and Depression Scale: significantly improved from baseline (anxiety *P* < 0.001, depression *P* < 0.05) Pittsburgh sleep quality index did not significantly improve from baseline (ns)
De Roest et al. [15], 2013	GSRS: a significant positive change in almost all the reported symptoms between baseline and follow-up (*P*s < 0.015) with exception of burping (*P* = 0.308), passage of mucus (*P* = 0.890), and feeling full even long after stopping eating (*P* = 0.051)	Not studied
Gravina et al. [19], 2020	NBD score: significant reduction compared to baseline (*P* < 0.05) Evaluation of abdominal pain using a 1 up to 10 levels scale of intensity: significant improvement compared to baseline (*P* < 0.05) Bristol scale: significant improvement of bloating, diarrhea, flatulence, and constipation was obtained both in terms of frequency and severity (all *P*s < 0.05)	Not studied
Harvie et al. [16], 2017	IBS-SSS: significantly reduced IBS-SSS from baseline to 6 mo. In group I (*P* < 0.05) and group II (*P* < 0.01) LFD is most effective for patients with IBS-D	IBS-QOL: significantly improved for group I from baseline to 6 mo (*P* < 0.05)
Kortlever et al. [17], 2019	GSRS: significant improvements in all GI symptoms (*P*s *<* 0.001), with the exception of constipation (*P* = 0.501) and nausea (*P* = 0.098)	IBS-QOL: total QOL significantly improved (*P* < 0.001) and all domains significantly improved from baseline, except for the “sexual” (*P* = 0.055) and “food avoidance” (*P* = 0.270) subdomains Subjective Vitality Scale: significant increase (*P* = 0.032), Happiness Measures: significant increase (*P* = 0.035) State-Trait Personality Inventory: significant reductions in depression (*P* = 0.01) and anxiety (*P* = 0.007) Fatigue Impact Scale: significant reductions compared with baseline (*P* < 0.003) Karolinska Sleep Questionnaire: no significant improvement from baseline (*P* = 0.239) Improvement in GI symptoms was significantly correlated with improvements in QOL (*r* = −0.513, *P* < 0.001)
Maagaard et al. [10], 2016	IBS-SSS: at follow-up overall median IBS-SSS score at follow-up was 211 (range: 16–487), no baseline data to compare it with Visual analog scales: the proportion of patients experiencing full effectiveness was 29% in IBS group. 37% of patients with IBS became asymptomatic while following the diet. The diet showed greatest effect on bloating (82%) and abdominal pain (74%) BSC: the proportion of patients producing normal stools significantly increased, with 41% in the IBS group (*P* < 0.001), particularly in the diarrhea group (*P* < 0.001) Copenhagen disease course: significant reduction in patients with a chronic continuous disease course (*P* < 0.001) along with a significant increase in patients with a mild indolent disease course (*P* < 0.001)	At follow-up, the median IBS-QOL score was 75 (range: 37–145) for the IBS group Most patients reported good quality of life Significant association (*P* < 0.001) between a good QOL and normal stool type after dietary treatment
Nawawi et al. [20], 2020	Symptom evaluation form: significantly improved all IBS symptoms (*P*s *<* 0.0001), except for heartburn (*P* < 1.0) BSC: approximately half of the patients had improved stool consistency, that is, Bristol stool type 3 and 4 (61% for patients with initial Bristol stool of types 5–7 and 47% for patients with initial Bristol stool of types 1–2) at 3-mo implementation. Most patients were satisfied with their overall symptoms’ improvement (76%, *n* = 31/41 at 12 mo)	Not studied
O’Keeffe et al. [12], 2018	GSRS: significant improvement in abdominal pain (*P* < 0.001), bloating (*P* < 0.001), flatulence (*P* < 0.001), incomplete evacuation (*P* = 0.007), and lethargy (*P* = 0.001) Global symptom question: compared with baseline, 57% of patients reported satisfactory symptom relief (*P* = 0.003) BSFS: significant reduction in the proportion of patients reporting abnormal stool frequency (*P* < 0.001) and abnormal stool consistency (*P* = 0.001)	Appointments with GP or GI doctors: no significant differences compared with an HD group (GP, *P* = 0.431, GI, *P* = 0.390) Medication use: significantly more AdLFD patients ceased medication (*P* = 0.048) compared with HD group Work absence: no significant differences in the number of days absent from work between groups taking ≥3 d off work in the last 12 mo because of to GI symptoms (*P* = 0.775)
Peters et al. [13], 2016	GI visual analog scale which is part of the IBS-SSS: significant improvements in overall GI symptoms (*P*s *<* 0.001). All individual symptoms (pain, bloating, wind, stool consistency) significantly improved (*P*s *<* 0.0001) except for nausea (ns)	IBS-QOL: significantly improved from baseline (*P* < 0.0001) State-Trait Personality Inventory state and trait anxiety and depression did not significantly improve (ns). Hospital Anxiety and Depression Scale: anxiety significantly improved (*P* = 0.037), depression did not significantly improve (ns)
Rej et al. [2], 2021	GSRS: of the 205 participants who completed the study, 74 of these participants (36%) had baseline symptom data available, collected as part of their routine dietetic care. In this subset of individuals, the proportion with moderate or severe symptoms was significantly lower at long-term follow-up vs. baseline for all symptoms studied (*P* < 0.03), with the exception of nausea (*P* = 0.05) BSFS: proportion of individuals with abnormal stools was significantly lower than the baseline (*P* < 0.01)	Not studied
Seamark et al. [18], 2021	GSRS: all symptoms significantly less frequently (*P*s < 0.001), with exception of heartburn and acid regurgitation Global symptom question: 55% satisfaction with gut symptom relief at long term compared with baseline (*P* < 0.001) BSFS: normal stool consistency significantly improved (*P* < 0.001) and normal stool frequency significantly (*P* = 0.005)	Appointments with GP or GI doctors: significantly decreased from baseline (GP visits *P* < 0.001, GI visits *P* = 0.002) Mediation use: no significant decrease from baseline (*P* = 0.034) (*P* < 0.01 considered to be statistically significant in this study)
Staudacher et al. [14], 2022	IBS-SSS: reduction in total score from baseline (median 227, IQR 99) and long term (154, 89; *P* < 0.001) GSRS: significant improvements in abdominal pain (*P* < 0.001), borborygmi (*P* = 0.006), bloating (*P* = 0.006), flatulence (*P* = 0.004), and incomplete evacuation (*P* = 0.014). Global symptom question: 67% of patients reported adequate relief long term compared with baseline (*P* = 0.039), 28% had such symptom improvement that they no longer met Rome III criteria BSFS: firmer stool consistency long term (*P* < 0.001) and lower stool frequency (*P* = 0.038)	IBS-QOL: significantly improved (*P* < 0.001), except for “food avoidance” (*P* = 0.377) and “sexual” (*P* = 0.384) SF-36: only pain subscale significantly improved (*P* = 0.011) Medication use: medication intake had remained stable for the majority of patients in the past 12 mo (16/18, 89%)
Weynants et al. [22], 2020	IBS-SSS: Less severe abdominal pain for patients still on LFD than patients who stopped or never started LFD (*P* = 0.044)	IBS-QOL: no significant difference in IBS-QOL between participants that no longer followed the diet and participants that still followed the diet (*P* = 0.639)

Abbreviations: AdLFD, adjusted low–fermentable oligosaccharides, disaccharides, monosaccharides, and polyols diet phase; BSFS, Bristol stool form scale; BSC, Bristol stool chart; GI, gastrointestinal; GP, general practitioner; GSRS, gastrointestinal symptom rating scale; HD, habitual diet; IBS, irritable bowel syndrome; IBS-D, irritable bowel syndrome with diarrhea; IBS-SSS, irritable bowel syndrome symptom severity score; IQR, interquartile range; LFD, low–fermentable oligosaccharides, disaccharides, monosaccharides, and polyols diet; NBD, neurologic bowel dysfunction questionnaire; QOL, quality of life; SF-36, Short Form Health Survey; ns, not significant.

The most widely used questionnaires for assessing GI symptoms and severity include the IBS–symptom severity score (IBS-SSS) [23], which comprises 0–500 points increasing with severity, the Gastrointestinal Symptom Rating Scale [24], and the Bristol stool form scale (also known as BSC) [25]. Four studies that used the IBS-SSS questionnaire compared the data to baseline, and all these studies indicated clinically significantly improved symptoms (*P*s < 0.05 or >50 points change on the IBS-SSS scale) with long-term IBS [13,14,16,21]. Six studies used the Gastrointestinal Symptom Rating Scale questionnaire and compared the data to baseline, and all noted a significant reduction in IBS symptoms [2,12,14,15,17,18]. Three of these also asked participants if they were satisfied with relief of gut symptoms at the long-term follow-up and 55%–67% of patients were satisfied, which is a significant increase compared with baseline [12,14,18]. Six studies that used the Bristol stool form scale, BSC, or a bowel habit questionnaire, all indicated that participants reported significant improvements in normal stools and bowel habits at the final evaluation compared with baseline [2,10,12,18,19,21]. Two studies used an unspecified symptom evaluation form or a neurologic bowel dysfunction questionnaire [26], and both reported significantly improved symptoms [19,20]. Some studies reported GI symptoms that did not significantly improve, including constipation and nausea [17], burping, the passage of mucus, feelings of satiety [15], heartburn and acid regurgitation [18,20], and nausea [13]. One study measured changes of IBS disease course using the Copenhagen IBS disease course questionnaire [27] and found a significantly improved disease course [10] at follow-up from baseline. Improvement in GI symptoms was significantly correlated with improvements in QOL [17].

#### QOL

Common questionnaires used to measure health-related QOL for patients with IBS are the IBS-QOL [28], the Short Form Health Survey (SF-36) [29], or specific questionnaires that measure anxiety, depression, happiness, vitality, or fatigue. Five studies using the IBS-QOL showed significant improvement in all QOL domains at final evaluation compared with baseline [13,14,16,17,30], with the exceptions of “sexual” and “food avoidance” [14,17]. An RCT using the SF-36 indicated that only the pain subscore significantly improved [14], while a prospective cohort study using the SF-36 showed that pain, vitality, social activity, and physical and mental health indexes significantly improved from baseline [21]. Two prospective cohort studies showed significant improvement in specific anxiety and depression questionnaires [17,21], whereas an RCT showed that only anxiety significantly improved in the Hospital Anxiety and Depression scale [13]. The prospective cohort study by Kortlever et al. [17] showed significant improvements in vitality and happiness, and significant reductions in fatigue from final evaluation to baseline.

For healthcare usage, measured by frequency of IBS appointments with a general practitioner or GI doctor, 1 prospective cohort study showed significant decreases at final evaluation compared with baseline [18], whereas another prospective cohort study indicated no significant differences compared with an HD group [12]. For IBS medication use, 2 prospective cohort studies showed no significant changes in medication use [14,18], whereas a third prospective cohort study found that significantly more patients ceased medication and fewer started medication at final evaluation compared with an HD group [12]. Additionally, this prospective cohort study also indicated no significant differences in the number of days absent from work due to GI symptoms between the Adjusted LFD and HD groups [12]. Two studies showed a positive association between symptom relief and QOL [10,17].

### Safety

Despite the long-term goal of an LFD to return to a minimally restrictive diet while maintaining maximum benefits, the potential for negative long-term effects regarding dietary adequacy, weight, body composition, and gut microbiome compositions presents concerns [14,21]. Safety results are summarized below and in Table 3.

**TABLE 3:** Safety

Study	Dietary intake	Weight and body composition	Microbiota and metabolic products
Ankersen et al. [30], 2021	Not studied	Data collected but results not included in article	LFD nonresponders tended to have a reduced diversity, responders did not *Faecalibacterium prausnitzii* significantly reduced by LFD and restored by reintroduction of high-FODMAP foods (*P* not provided)
Bellini et al. [21], 2020	Not studied	BIVA: no significant changes compared with baseline (ns) Weight: no significant changes compared with baseline (ns)	Not studied
Harvie et al. [16], 2017	FODMAP specific food-frequency questionnaire: food consumption and fiber intake decreased during the strict phase but increased again to levels similar to preintervention. Energy, protein, fat, carbohydrate, fiber, and calcium intakes exceeded the New Zealand estimated average requirements at long-term follow-up	Not studied	No differences in microbiota diversity; however, results are inconclusive because of study error
O’Keeffe et al. [12], 2018	CNAQ: nutritional adequacy of energy, macro and micronutrients including fiber was not compromised for either the AdLFD group or the HD group at long-term follow-up compared with the UK DRVs, and there were no significant differences between the groups except that folate and vitamin A were higher for the AdLFD. At least 95% in both groups met the appropriate DRV for energy and the majority of nutrients. Total carbohydrate and calcium intakes were not different between groups. Total FODMAP intake was significantly lower for the AdLFD group vs. HD group. Lactose and high-FODMAP foods were significantly lower while low-FODMAP foods were significantly higher for the AdLFD vs. HD group. Similar intakes for high and low-FODMAP cereals and grain between groups, but AdLFD had a higher intake of low-FODMAP bread and a lower intake of high-FODMAP pasta compared with the HD group. The AdLFD group had a higher intake of low-FODMAP milk, low-FODMAP vegetables, and a lower intake of fats and oil than the HD group. The AdLFD group ate significantly less garlic and fewer people in this group ate high-fructan foods compared with the HD group	Weight: 42% of patients reported weight reduction from baseline at long-term follow-up. However, these weight data were self-reported at long term, so must be interpreted with caution	Not studied
Rej et al. [2], 2021	CNAQ: pLFD individuals failed to meet total energy and the majority of macronutrient DRVs at long-term follow-up compared with the UK DRVs but mean nutritional intake and total and specific FODMAP group intake did not differ between the pLFD group vs. HD group. Only 41% of those on pLFD and 39% of those on their HD met dietary fiber requirements per UK DRVs at long-term follow-up	Not studied	Not studied
Staudacher et al. [14], 2022	FODMAP intake: no significant difference from baseline except for sorbitol (*P* = 0.028). Fiber intake did not change (*P* = 0.349). Iron (*P* = 0.005), energy (*P* = 0.043), carbohydrate (*P* = 0.039), protein (*P* = 0.011), and fat (*P* = 0.048) were significantly lower in the long term compared with baseline	Weight: no significant changes compared with baseline (*P* = 0.711)	No significant changes in Bifidobacteria spp. Significantly lower relative abundances of Bacteroides spp. (*q*∗ = 0.022), *Faecalibacterium prausnitzii* (*q*∗ = 0.001), and *Ruminococcus bromii* (*q*∗ = 0.024) ∗*q* = *p* with adjustment for false detection rate. However, the absolute abundance of these 3 microbiotas did not change, suggesting that there was not a specific change in these bacteria, but possibly a change in other bacteria. Significantly lower concentrations of total SCFA (*P* = 0.004), acetate (*P* = 0.002), propionate (*P* = 0.001), and butyrate (*P* = 0.035)

Abbreviations: AdLFD, adjusted low–fermentable oligosaccharides, disaccharides, monosaccharides, and polyols diet phase; BIVA, Bioelectrical Impedance Vector Analysis; CNAQ, Comprehensive Nutrition Assessment Questionnaire; DRVs, daily reference values; FODMAP, fermentable oligosaccharides, disaccharides, monosaccharides, and polyols; HD, habitual diet; LFD, low–fermentable oligosaccharides, disaccharides, monosaccharides, and polyols diet; pLFD, personalized low–fermentable oligosaccharides, disaccharides, monosaccharides, and polyols diet phase; SCFA, short-chain fatty acids; ns, not significant.

#### Dietary intake

Overall intakes of energy, macronutrients, and micronutrients were examined within the included studies. One study that used the Comprehensive Nutrition Assessment Questionnaire [12] and another that used the food-frequency questionnaire [16] to assess nutrient intake showed that dietary intake was nutritionally adequate at long term compared with national recommendations. However, 1 additional study found that dietary intake did not meet national recommendations long term, but that these intakes did not differ between the personalized LFD compared with HD groups [2].

#### Weight and body composition

Two prospective cohort studies showed no significant changes in weight from baseline [14,21]. Bioelectrical impedance vector analysis parameters, measurements to evaluate body composition, hydration, and phase angle, did not significantly change at long-term evaluation compared with baseline [21]. Phase angle is a sensitive early marker for energy intake for patients at nutritional risk; however, it is not yet validated for this purpose [21].

#### Gut microbiota

The human gut microbiome is a highly diverse group of trillions of microorganisms that live in the human digestive system [31]. The alteration of the microbiota balance, which includes changing its diversity, is associated with many diseases [31], which has led to concerns regarding potential changes in the microbiota while on an LFD. There are also observations regarding an imbalance of the microbiota playing a part in the pathogenesis of IBS, with the development of IBS following gastroenteritis, low-grade mucosal inflammation, abnormal fermentation, and evidence of small bowel bacterial overgrowth [32]. Two studies analyzed stool samples to measure changes in the gut microbiota from baseline to the final evaluation time point [14,30] and found no differences in long-term gut microbiota, but did find the absolute abundance of the stool short-chain fatty acids (SCFAs) acetate, propionate, and butyrate decreased [14]. It is important to note that fiber and total FODMAP intakes were maintained between baseline and long-term evaluation [14]. Additionally, nonresponders tended to have a reduced microbiome diversity compared with responders [30].

### Sustainability

Concerns and findings surrounding long-term diet sustainability, including adherence and acceptance, are summarized below and in Table 4.

**TABLE 4:** Sustainability

Study	Adherence	Acceptance
Ankersen et al. [30], 2021	FARS (score of 22 or higher of 25 considered adherent): 8 of 12 (67%) participants completed the reintroduction to high-FODMAP foods and developing an individualized diet at 1-y follow-up	Patients reintroduced a median of 14.5 high-FODMAP foods, of which participants categorized a median of 7 foods as green (i.e., not symptom-triggering foods), 5 foods as yellow (i.e., mild to moderate symptom-triggering foods), and 2 foods as red (i.e., severe symptom-triggering foods). Common among symptom-triggering foods were wheat and rye bread, pasta, pointed cabbage, onion, garlic, leek, broccoli, green peas, cauliflower, kidney beans, chickpeas, sweet potatoes, avocado, mushrooms, apples, and apple juice
Bellini et al. [21], 2020	FARS (≥80% of 20 points was considered adherent): excellent adherence during strict phase, but adherence slightly declined long term. Adherence assessed at T3 compared with after strict phase appeared to have decreased just below the cut-off value (*P* < 0.0001)	Acceptability: 51.2% of patients during the AdLFD reported that they spent more time for shopping compared with their HD (*P* < 0.003). 39% during the AdLFD reported that they spent more time for cooking (*P* < 0.01). During the AdLFD, there was a slight, but not significant, reduction of time spent for shopping and cooking compared with the LFD. Patients reported that AdLFD (58.5%) were more expensive in comparison with their HD (*P* < 0.0003). The AdLFD was judged to be slightly, but not significantly, cheaper than the LFD. 58.5% during the AdLFD reported increased difficulty eating out at restaurants compared with the HD (*P* < 0.03, respectively). During AdLFD, 76.6% of the patients found increased difficulty in eating out at friends (*P* < 0.00001) and 65.8% found more difficulty eating during travel (*P* < 0.0002). Patients found the AdLFD (29.2%) less tasty and enjoyable than their HD (*P* < 0.02) Food-related QOL: The conditions of patients’ life regarding food were better with the AdLFD (56.1%) in comparison with their HD (15.0%) (*P* < 0.007). Patients were more satisfied with food and meals in daily life during AdLFD (70.7%) compared with the HD (45.0%) (*P* < 0.05). The wish that their meals were a much more pleasant part of their life was significantly higher with the HD (60.0%) compared with the LFD (29.2%) and AdLFD (33.9%) (*P* < 0.03 and *P* < 0.05, respectively). The feeling of “seeing problems with food” significantly decreased during the LFD (9.8%) and AdLFD (14.6%) in comparison with the HD (60.3%) (*P* < 0.0003 and *P* < 0.003, respectively)
De Roest et al. [15], 2013	Adherence questionnaire not specified. 76% patients described ongoing adherence to the diet. Adherence meant they followed the diet 50% of the time or more There was a significant positive correlation between adherence and improvement in bloating (*r* = 0.273, *P* = 0.011), abdominal pain/discomfort (*r* = 0.271, *P* = 0.010), flatulence/wind (*r* = 0.374, *P* = 0.0001), diarrhea (*r* = 0.310, *P* = 0.007), constipation (*r* = 0.296, *P* = 0.017), and energy levels (*r* = 0.271, *P* = 0.013)	72.1 % patients were satisfied with their overall symptoms 89.5% thought that the written information was easy to understand. 60% patients stated that the diet was easy to follow, 65.1% could easily find suitable products, and 43.5% were able to incorporate the diet easily into their life. The overall taste was liked by 54.7% patients, although 24.4% thought that the diet was too expensive. 18.2% believed that simply being given a list of foods to avoid would have been as effective as seeing the dietitian for a consultation whereas 44.6% patients would have liked to have seen the dietitian for a further follow-up appointment. Written information and dietitian consultation were ranked the highest, whereas the support of family and friends, low-FODMAP cookbooks, and online information were thought to be less important
Maagaard et al. [10], 2016	FARS (≥80% of 20 points was considered adherent): adherence rate for IBS group is unclear. Adherence was the highest with regard to remembering to follow the diet and not taking breaks, and poorest when asked if patients followed the diet “by the book” and without making their own modifications	Questionnaire used VAS scales (≥60% of 100 points was considered as satisfaction with dietary treatment): Satisfaction with dietary management was seen in 70% of patients with IBS Wheat, dairy products, and onions were the foods most often not reintroduced by patients
Nawawi et al. [20], 2020	Adherence was recorded by a combination of patient food and symptom diary and dietary recall on detailed questioning from the dietitian. 50% (7/14) continued to restrict some high-FODMAP foods, but not all the high-FODMAP foods Best symptom improvement was seen in those who were fully adherent to the FODMAP diet	Not studied
O’Keeffe et al. [12], 2018	4-point Likert scale: “continued strict low-FODMAP diet,” “reintroduced high-FODMAP foods to tolerance,” “continued low-FODMAP diet 50% of the time,” “returned to habitual diet.” The “adapted FODMAP” group included the first 3 of these. 82% patients continued to follow an “adapted FODMAP” diet, whereas 18% returned to a “habitual” diet	There were no significant differences between the AdLFD compared with the HD group for the majority of components of dietary acceptability, except 86% patients in the “adapted FODMAP” group reported that their diet was more expensive than before following the diet, compared with only 8 (42%) patients in the “habitual” group (*P* < 0.001). The “adapted FODMAP” group reported increased difficulty eating out at restaurants compared with the “habitual” group (66 [79%] vs. 11 [58%] *P* = 0.013), eating at family and friends’ houses (61 [72%] vs. 9 [48%] *P* = 0.009), and eating when traveling (63 [76%] vs. 9 [48%] *P* = 0.014). However, there were no significant differences for any of the components of food-related QOL between groups
Rej et al. [2], 2021	76% patients still following LFD, with the remainder were either on the strict reduction phase of the low-FODMAP diet (6%), returned to HD (9%), gluten- or wheat-free diet (4%), lactose-free diet (2%), or another diet (3%). The majority of participants on the pLFD at long term had minor lapses (70%), with strict adherence noted in 26% of individuals, and major lapses in 5% of individuals Adequate relief of symptoms was noted in 68% who were strictly adherent or had minor lapses, compared with 13% of individuals who had major lapses	Nutrition-related QOL: compared with the HD group, significantly more people on the pLFD found that it took extra time to shop (*P* < 0.01), it cost more (*P* < 0.01), it was more difficult eating out with friends and family and at restaurants and while traveling (all *P*s < 0.01), which made it more difficult to follow the diet (*P* < 0.01) and less easy to incorporate into life (*P* < 0.01). However, they had greater food and meal satisfaction (*P* = 0.03) and fewer saw obstacles when thinking about the next meal (*P* = 0.03) Patients had multiple dietary requirements when eating out, and 80% of those on pLFD consumed specific “free-from” products such as gluten- or wheat-free products
Weynants et al. [22], 2020	Self-developed adherence questionnaire: 80% participants were still following a diet in which certain FODMAP-rich food types were being avoided. 19% patients no longer avoided FODMAP-rich food types, and 1% of participants did not start the diet because of pregnancy	89% of participants were satisfied that they follow or had followed the diet

Abbreviations: AdLFD, adjusted low–fermentable oligosaccharides, disaccharides, monosaccharides, and polyols diet phase; FARS, fermentable oligosaccharides, disaccharides, monosaccharides, and polyols adherence report scale; FODMAP, fermentable oligosaccharides, disaccharides, monosaccharides, and polyols; HD, habitual diet; IBS, irritable bowel syndrome; LFD, low–fermentable oligosaccharides, disaccharides, monosaccharides, and polyols diet; pLFD, personalized low–fermentable oligosaccharides, disaccharides, monosaccharides, and polyols diet phase; QOL, quality of life; VAS, visual analog scale.

#### Adherence

Adherence is the degree to which individuals followed the diet at the final evaluation time point. Adherence was measured with the FODMAP adherence report scale, developed by Maagaard et al. [10], where a total score of 20 or 22 of 25 points is considered adherence to the diet, other self-developed adherence questionnaires, which defined adherence as following the diet 50% or more, or with an unspecified adherence questionnaire which noted adherence as avoiding some FODMAP-rich foods. Using these various questionnaires, 6 studies of all study design types reported adherence rates ranging from 50 to 82% [2,12,15,20,22,30]. Adherence results did not vary regarding study design, study duration, subject populations, follow-up time point, or country context. There was very high variability in how adherence was defined and measured, which could greatly impact reported adherence rates. Therefore, this range should be used with caution, and future research should attempt to standardize a definition and measurement method for adherence whenever possible.

Remembering to follow the diet, not taking breaks, and making individual modifications to the LFD increased adherence in a retrospective study [10]. Reasons for nonadherence included the inability to adhere due to time management, the absence of an effect, no longer experiencing symptoms, too complicated to follow, too expensive, bland in taste, comorbidities, or other reasons [10]. There was a significant positive correlation between adherence and improvement in some IBS symptoms [15], and better adherence to an LFD was associated with improved symptom relief [2,20].

#### Acceptance

Diet acceptance examines how well participants can incorporate an LFD into their everyday lives. Common difficulties participants noted with the LFD were higher expense, eating out at restaurants, at family and friends, and when traveling [2,12,15,21]. Other difficulties noted by participants were that they spent more time shopping [2,21], found the diet less tasty and enjoyable [21], and spent more time cooking [2], which made the diet more difficult to incorporate into life [2].

Despite diet challenges, the LFD was acceptable to many participants. Four studies examined study participant satisfaction with the LFD and found that 70%–89% of study participants were satisfied with the LFD for IBS management [10,15,21,22]. A large multicenter retrospective study found that participants reported greater food and meal satisfaction, and fewer obstacles when thinking about the next meal [2]. Although 2 prospective cohort studies found that the majority of participants indicated the diet was easy to follow and incorporate into their lives, liked the overall taste [12], and that the conditions of their lives regarding food were better, meals were a much more pleasant part of their lives, and “seeing problems with food” significantly decreased compared with their HD counterparts [21].

Patients modified their diets in specific ways over the long term to increase diet acceptability, such as consuming specific diet products like those that were gluten-free or wheat-free [2]. Wheat was found as both a common symptom-triggering food [30] and 1 of the foods most often not reintroduced by patients [10].

### Infographic

**FIGURE 1:**
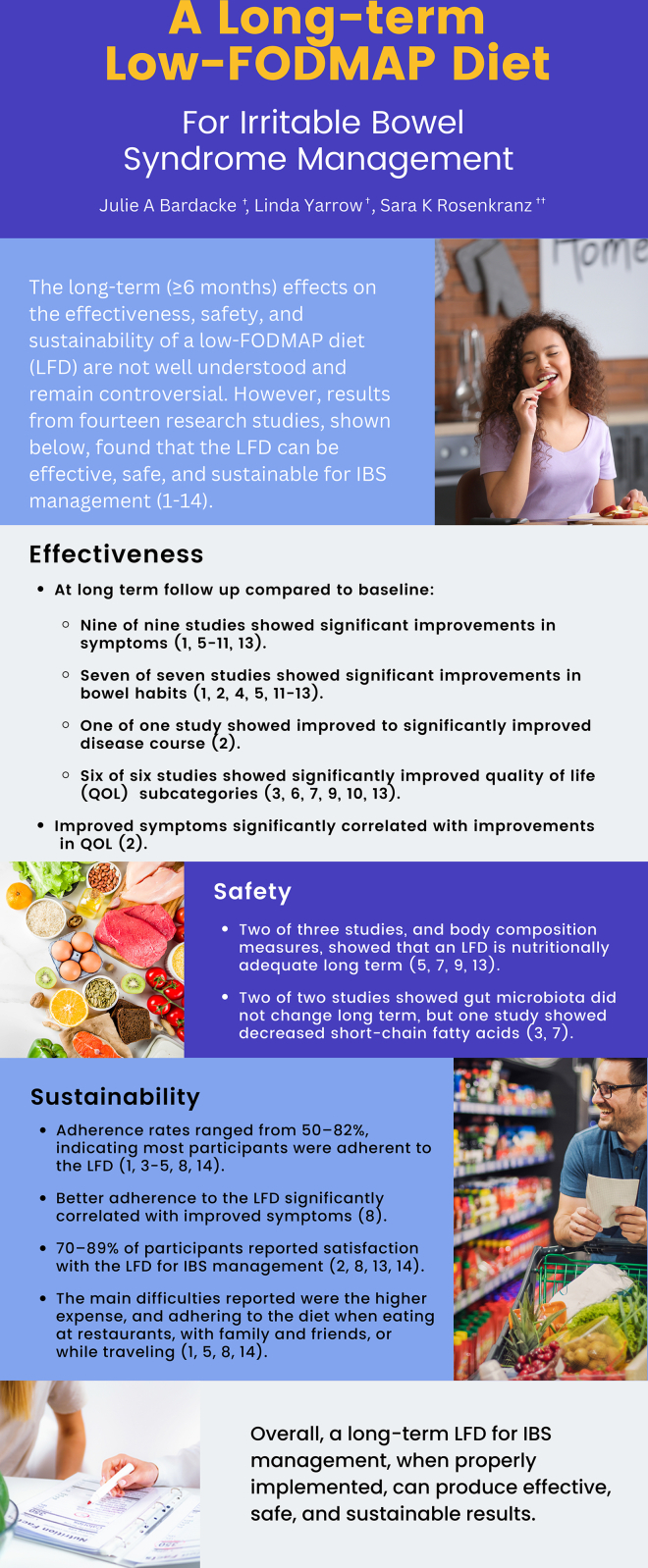

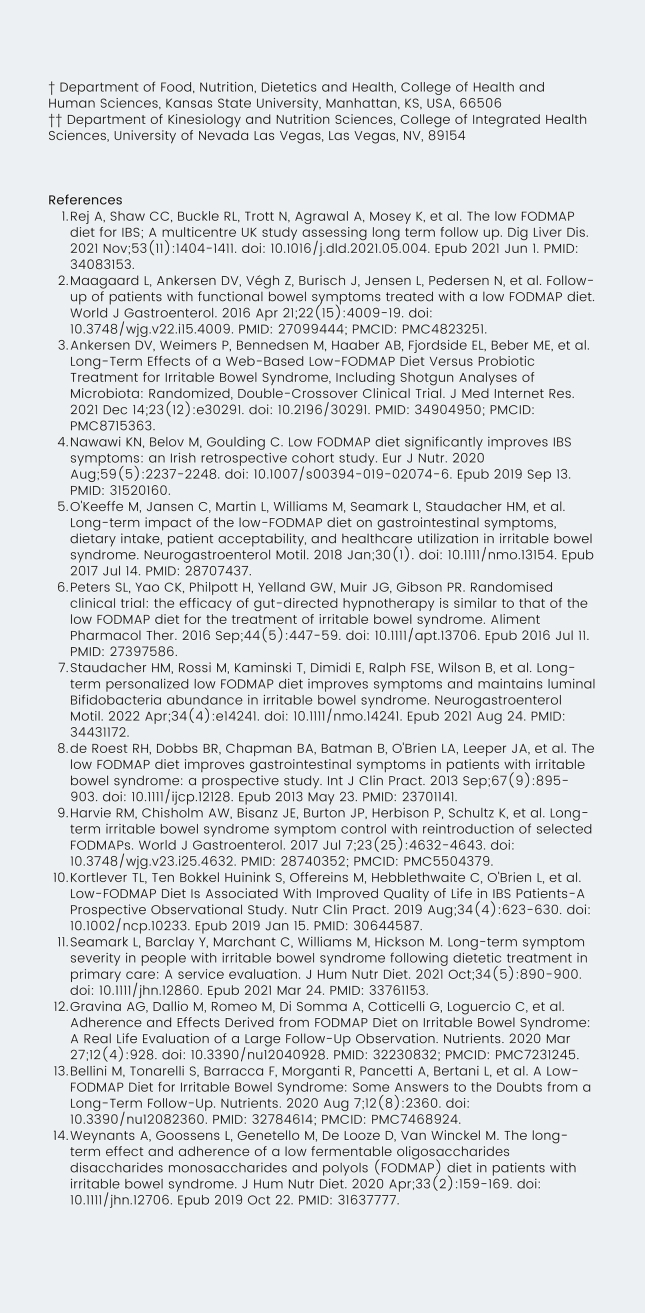
Long-term low–fermentable oligosaccharides, disaccharides, monosaccharides, and polyols diet (LFD) for irritable bowel syndrome (IBS) management infographic. Summary of the current review results shown as an infographic for dissemination of information to registered dietitian nutritionists and other health professionals, for use in providing LFD education to patients with IBS. The infographic addresses the effectiveness, safety, and sustainability of a long-term LFD and includes topics that a registered dietitian nutritionist may potentially focus on when providing LFD education. FODMAP, fermentable oligosaccharides, disaccharides, monosaccharides, and polyols; QOL, quality of life.

Since the conception of the LFD, research pertaining to this diet has increased, making it more time-consuming for healthcare professionals to keep up with the newest research. An infographic provides a rapid and effective way to disseminate up-to-date scientific research for healthcare professionals such as RDNs with time constraints [33,34]. The infographic shown in Figure 1 summarizes the current review results for dissemination of information to RDNs and other health professionals for use in providing LFD education to patients with IBS. The infographic addresses the 3 areas covered in the current review and includes topics that an RDN may potentially focus on when providing LFD education. The resultant infographic was checked using the Developing & Assessing Nutrition Education Handout checklist created by the Academy of Nutrition and Dietetics Foundation, to assess its quality as a nutrition education handout. The infographic adheres to at least 18 of 21 rated points, which indicates that it can be considered a quality nutrition education handout.

## Discussion

The aims of the current study were to review the published research on the effectiveness, safety, and sustainability of an LFD for patients with IBS, and to develop an infographic for disseminating findings to outpatient RDNs and other healthcare professionals who work with patients with IBS. Overall, the current review indicates that an LFD is effective for improving IBS symptom relief. Nine of 9 studies indicated clinically significantly improved symptoms [2,[12], [13], [14], [15], [16], [17], [18],^21^], 7 of 7 studies indicated significant improvements in bowel habits compared with baseline [2,10,12,[18], [19], [20], [21]], and 1 study indicated a significantly improved disease course [10], compared with baseline. There were fewer significant improvements reported for some upper GI symptoms and constipation [13,15,17,18], which might be due to the osmotic effect of FODMAPs in the lower bowels, making the LFD less effective in the upper GI region [15] and for patients with constipation-predominant IBS[17].

Long-term QOL significantly improved in subcategories for 6 of 6 studies [13,14,16,17,21,30]. However, food avoidance did not significantly improve [17] or decreased [14], which is important to highlight as there is evidence that people with GI disorders who undergo dietary changes may be at increased risk for disordered eating behaviors and may experience more food-related anxiety [35]. A 2021 systematic review conducted by Peters et al. [36] indicated that disordered eating prevalence rates for individuals with GI disorders were 13%–55%, and prevalence was higher in those with disorders of gut-brain interaction such as IBS, than in those with organic GI disorders. However, these rates might be similar to those found in other dietary-controlled health conditions that require consistent monitoring of food intake [35]. It remains challenging to identify patients who are at-risk patients for eating disorders [36], but healthcare professionals working with patients of IBS must stay vigilant when working with those on the LFD to monitor for signs of disordered eating [35].

The QOL in those with IBS is associated with other factors such as lost work productivity and higher healthcare and medication usage, but these associations are not completely understood, and might be influenced by personal coping ability and confidence in healthcare [37]. In the current review, the impact of an LFD for patients with IBS on healthcare and medication usage, as well as impacts on work attendance, showed varied results, and therefore, need further research to determine the effects of these factors. The current review found a significant association between good QOL and normal stool type after dietary treatment [10], and improvement in GI symptoms was significantly correlated with improvements in QOL [17], which aligns with numerous studies that have shown that health-related QOL improves when symptoms of the disease are reduced [38].

Overall, the current results indicate that the LFD is generally safe over the long term. Two of 3 studies noted that nutritional adequacy was not compromised over the long term after FODMAPs were reintroduced [12,16]. However, these results must be interpreted with caution, as FFQs used to assess nutrition intake might not be as accurate as prospective food records [39]. One study did note that individuals on long-term LFD and those who returned to their HD, failed to meet total energy and the majority of macronutrient needs, suggesting that dietary behaviors of patients with IBS, whether they are under treatment or not, might have lower intakes for certain nutrients [2]. Body composition measures also showed that nutritional adequacy was not compromised [21], and body weight remained the same [14,21] or decreased, possibly due to participants following overall healthier eating habits [12].

Two of 2 studies showed gut microbiota did not change after reintroduction of FODMAPs into the diet [14,30]. Of note, LFD nonresponders had reduced microbiota diversity compared with responders, an area that requires further research, but can possibly shed light on why LFD was not equally effective for all patients [30]. The biggest concern noted among the included studies, was the decrease in SCFAs shown in 1 study, despite the reintroduction of dietary fiber, which is fermented in the colon and produces SCFAs [14]. While it is not clear what effects this reduction would have on long-term health, these results are of potential concern given the beneficial regulatory role of SCFA in gut function, such as intestinal motility [14]. The changes in SCFAs may be due to a change in total and specific types of carbohydrates consumed, variable stool volume, and colonic transit time [14].

Overall, the current results indicate the LFD is sustainable. Adherence rates ranged from 50 to 82% [2,12,15,20,22,30], indicating that most patients who maintained an LFD over the long term, were considered to be adherent to the LFD in the majority of studies. Even though adherence rates decreased slightly after the initial LFD phase, any chronic therapy treatment tends to have decreased adherence over time, even when positive results are occurring [21]. Although the LFD diet is restrictive in the beginning, the reintroduction and personalization processes are essential parts of the LFD, and may positively impact adherence.

Participants accepted the diet, as 70%–89% of study participants were satisfied with the LFD for IBS management [10,15,21,22]. However, participants found the LFD challenging. Some of the main difficulties are that LFD can be more expensive than a non-LFD diet, and more difficult to comply with when eating at restaurants, with family and friends, or while traveling [2,12,15,21]. While many participants did not find the diet easy to incorporate into their lives, most patients remained adherent, which suggests that perceived difficulties are outweighed by improvements in symptoms [15]. Additionally, difficulties reported when following an LFD are not unique to the LFD, as the same difficulties have been reported studies of other diets [2]. To ease diet challenges, participants also modified their diets by consuming specific gluten-free or wheat-free products, some of which might be more expensive or unavailable when eating out [2]. While wheat was found to be a common symptom-triggering food, more research is needed to identify which wheat components (i.e., FODMAPs, gluten, wheat germ agglutinins, and alpha-amylase trypsin inhibitors) are the main culprits [2].

The LFD can be confusing and complex to learn and follow, and challenging to teach. A healthcare professional can help screen patients to determine if the LFD is a suitable treatment, provide support and guidance, such as regarding awareness of food additives and reading food labels [39], manage expectations, monitor adherence and results, and help patients stop the diet if symptoms are not improved or there are negative side effects. Healthcare professionals can also help to ensure nutritional adequacy by optimizing nutrient intake and monitoring dietary quality. Although not included in the results, several studies emphasized that using a trained RDN to follow the LFD can help in many ways and may help increase the likelihood of success. Specialist RDNs were widely recommended as the primary provider of LFD counseling [11,14,39,40]. However, there are challenges in finding specialist RDNs, which might be mitigated by group classes and online courses, or using supplemental applications such as the Monash University FODMAP diet application [41] for support in finding the FODMAP content of foods. In addition, a web-based application for tracking and monitoring symptoms [30] may be helpful for patients and healthcare providers.

The current review had several strengths and limitations. Strengths include that the review was comprehensive, used multiple electronic databases, included high-quality studies, and was up-to-date. The synthesis of the results of included studies led to the creation of an infographic to be used for dissemination to, and by RDNs, and other healthcare professionals. While not the aim of the review, limitations include that it was not a systematic review, and did not include a quantitative evaluation or meta-analysis. Overall, we aimed to include the highest quality available published research. However, most studies were prospective or retrospective, which decreases the overall strength of the current review. An additional potential limitation is that some relevant studies that met eligibility criteria due the search terms, such as “long term,” may have been missed. However, the original search did yield several studies that were <6 mo in duration, and we believe the search strategy yielded relevant studies, particularly with the additional steps of citation matching and searching in the reference lists of included studies.

Based on reported participant completion ranges from 6 studies, 17%–81% of participants dropped out or were excluded after initial enrollment, which might increase risk of bias because those who did not respond or participate may have had worse symptom outcomes than those who did. The high adherence rates might also be attributed to participation bias, meaning that people who adhered more to the diet also responded more often to the final evaluation. Additionally, most studies had sample sizes of <100 participants, possibly decreasing the power of these studies. Studies used statistical methods to account for potential bias and small sample sizes by including intention-to-treat, per-protocol, last observation carried forward, or calculating power to determine the minimum number of questionnaires that needed to be completed [[12], [13], [14], [15], [16], [17]].

Assessment methods and questionnaires varied between studies. Some questionnaires may be more prone to recall bias than other more objective measurements, particularly when used in retrospective study designs. Also, in most studies, an RDN delivered the dietary education and collected data, which might lead to biased results because patients wanted to assist the RDN, or the RDN was biased in some way. Although double-blinded RCTs may not be feasible, it would be possible to have a blinded research assistant do assessments in order to minimize the potential for researcher bias. It is important for future studies to use standardized and validated assessment methods and questionnaires to move this area of research forward.

## Conclusion

Following a long-term LFD for IBS management can be effective, safe, and sustainable. This type of diet, when properly implemented, can effectively improve IBS symptoms and QOL, without sacrificing nutritional adequacy, weight, body composition, or gut microbiota composition. Despite challenges with social eating and higher food costs, many patients adhered to and accepted the diet. More long-term studies are needed to confirm these findings, ideally RCTs with larger sample sizes and objective evaluation tools. However, thus far, the current results can be interpreted with caution, indicating that personalized LFD for IBS management can produce overall positive and effective, safe, and sustainable results.

## Author contributions

The authors’ responsibilities were as follows – JAB: conceptualized and designed the review with input from LY; JAB: wrote the draft manuscript and analyzed the data; LY, SKR: critically revised the manuscript for important intellectual content; SKR: had primary responsibility for the final content; and all authors: read and approved the final manuscript.

## Conflict of interest

The authors report no conflicts of interest.

## Funding

The authors reported no funding received for this study.
